# Wasp venom from *Vespa magnifica* acts as a neuroprotective agent to alleviate neuronal damage after stroke in rats

**DOI:** 10.1080/13880209.2022.2032207

**Published:** 2022-02-16

**Authors:** Hairong Zhao, Mei Wang, Xi Huang, Xiumei Wu, Huai Xiao, Fanmao Jin, Jiaming Lv, Jidong Cheng, Yu Zhao, Chenggui Zhang

**Affiliations:** a Yunnan Provincial Key Laboratory of Entomological Biopharmaceutical R&D, Dali University, Dali, PR China; b School of Medicine, Xiamen University, Xiamen, PR China; c National-Local Joint Engineering Research Center of Entomoceutics, Dali, PR China

**Keywords:** Ischaemic stroke, neuroinflammation, brain–blood barrier

## Abstract

**Context:**

Acute ischaemic stroke (AIS) is a major cause of disability and death, which is a serious threat to human health and life. Wasp venom extracted from *Vespa magnifica* Smith (Vespidae) could treat major neurological disorders.

**Objective:**

This study investigated the effects of wasp venom on AIS in rats.

**Material and methods:**

We used a transient middle cerebral artery occlusion (MCAO) model in Sprague-Dawley rats (260–280 g, *n* = 8–15) with a sham operation group being treated as negative control. MCAO rats were treated with wasp venom (0.05, 0.2 and 0.6 mg/kg, *i.p.*) using intraperitoneal injection. After treatment 48 h, behavioural tests, cortical blood flow (CBF), TTC staining, H&E staining, Nissl staining, TUNEL assay, immunohistochemistry (IHC) and ELISA were employed to investigate neuroprotective effects of wasp venom.

**Results:**

Compared with the MCAO group, wasp venom (0.6 mg/kg) improved neurological impairment, accelerated CBF recovery (205.6 ± 52.92 *versus* 216.7 ± 34.56), reduced infarct volume (337.1 ± 113.2 *versus* 140.7 ± 98.03) as well as BBB permeability as evidenced by changes in claudin-5 and AQP4. In addition, function recovery of stroke by wasp venom treatment was associated with a decrease in TNF-α, IL-1β, IL-6 and inhibition activated microglia as well as apoptosis. Simultaneously, the wasp venom regulated the angiogenesis factors VEGF and b-FGF in the brain.

**Conclusions:**

Wasp venom exhibited a potential neuroprotective effect for AIS. In the future, we will focus on determining whether the observed actions were due to a single compound or the interaction of multiple components of the venom.

## Introduction

Acute ischaemic stroke (AIS) is a major cause of disability and death; it is a serious threat to human health (de Rooij et al. 2013; Turner et al. 2013; Wu et al. 2019). It accounts for about 80% of all strokes (Su et al. 2021), and the percentage is gradually increasing. Within 8–10 min of ischaemic attack, the neurons in the ischaemic core area immediately undergo irreversible necrosis, and a salvageable penumbra, dominated by neuronal apoptosis, is formed around the ischaemic core area (Hakim 1987; Ginsberg and Pulsinelli 1994; Baron 1999; Zhang XM et al. 2010). The reason is that the focal ischaemia consists of the core necrotic area and penumbra. The cortical blood flow (CBF) in the core necrotic area drops below 25% of the normal, while that in the ischaemic penumbra remains above the threshold value due to the perfusion of collateral circulation, resulting in the loss of neuronal function and retention of structural integrity.

Recently, major therapeutic strategies for AIS focus on the restoration of CBF and survival of the ischaemic penumbra with thrombolytic drugs, such as recombinant tissue plasminogen activator (Levy et al. 2009; Wardlaw et al. 2012; Jauch et al. 2013; Xu et al. 2013). However, intravenous thrombolysis has a strict limitation of time-window; less than 3% of stroke patients can benefit from these interventions, and two-thirds of them still exhibit different degrees of disabilities (Hacke et al. 2008; Emberson et al. 2014). The subsequent reperfusion by thrombolytic therapy after AIS can also accelerate cerebral injury, resulting in encephaledema, cerebral haemorrhage and neuronal death. This phenomenon is called cerebral ischaemia/reperfusion (I/R) injury. The cerebral I/R injury is caused by various types of cellular stresses (Lo et al. 2003), including energy failure (Giaume et al. 2010; Pereda 2014), oxidative stress (Moskowitz et al. 2010), the elevation of the intracellular Ca^2+^ levels (Araki et al. 1992), release of excitatory neurotransmitters (Amara 1992), neuro-inflammatory responses (Sternberg 2006; Low et al. 2014) and apoptosis (Lipton 1999). The ischaemic penumbra progressively exacerbates in a few days after stroke. Therefore, the development of new sources remains a challenge for AIS.

Our previous results showed that the wasp venom extracted from *Vespa magnifica* Smith (Vespidae) had a protective effect on rheumatoid arthritis in rats (Gao et al. 2020). Additionally, compounds extracted from the wasp venom have also been used to treat major neurological disorders, including epilepsy (Mortari et al. 2005), Parkinson’s disease (Khalil et al. 2015), and Alzheimer’s disease (Thathiah and De Strooper 2011). The low molecular weight compounds extracted from *Polybia platycephala* Richards (Hymenoptera: Vespidae) wasps could block pentylenetetrazol-induced seizures (Mortari et al. 2005). Bradykinin extracted from wasp venom could protect against delayed neuronal death in post-ischaemic rat hippocampus (Danielisova et al. 2008, 2009). Accumulating evidence has also shown that bradykinin has effective anti-inflammatory properties and inhibits activated microglia by down-regulating TNF-α and IL-1β (Noda et al. 2007). These findings suggested that wasp venom might exhibit potential neuroprotective effects.

Our previous study has confirmed that four compounds, including 5-hydroxytryptamine, vespakin M, mastoparan M and vespid chemotactic peptide M, were purified and identified from wasp venom (Zhou et al. 2019). However, the effects of wasp venom on stroke have not been investigated yet. Therefore, this study investigated the effects of wasp venom on stroke using middle cerebral artery occlusion (MCAO) rat models and their underlying mechanisms. Our results systematically demonstrated the wasp venom as a neuroprotective agent, alleviating the functional recovery after stroke in rats.

## Materials and methods

### Animals

Adult male Sprague-Dawley rats, weighing 260–280 g, were obtained from Hunan Leske Jingda Experimental Animal Co., Ltd. (Animal certificate no.: SCXK (Xiang) k2013-0004). All the animal experiments were approved by the Institutional Animal Care and Use Committee (IACUC) of Dali University, China. All the rats were housed in a specific pathogen-free facility (SPF) under a 12 h light/dark cycle in a temperature-controlled environment (22–25 °C) with a humidity of 40–70% and free access to food and water.

### Determination of *Vespa magnifica* venom by high performance liquid chromatography (HPLC)

The wasp venom was provided by the National-Local Joint Engineering Research Centre of Entomoceutics, Dali, China. The quality control methods of all the tested samples are described in our previous study (Zhou et al. 2019). The HPLC method was used for quality control, the chromatographic conditions of which were as described previously (Zhou et al. 2019).

### Surgical procedure: cerebral ischaemia by MCAO

The rats in the sham group underwent the same surgery without ligating the arteries. MCAO surgery was performed as described previously (Longa et al. 1989; Kuge et al. 1995; Zhang et al. 2020). All the rats were deeply anaesthetized with 10% chloral hydrate (300 mg/kg, *i.p.*), and their internal common carotid artery (CCA) and external carotid artery (ECA) were gently separated. A 0.36-mm nylon suture with an L3600 silicon-coated tip (Guangzhou Jialing Biotechnology Co., Ltd., China) was carefully inserted into the internal carotid artery from the ECA to occlude the middle cerebral artery. After occluding for 1.5 h, the nylon suture was removed, and blood circulation was restored.

Physiological parameters were monitored as described previously (Wang et al. 2001). Blood pressure was evaluated by a blood pressure recorder (Softron, Japan). Arterial blood was analysed by a blood gas analyser (GEM Premier 3000, USA).

The body temperature was maintained at the normal range (36.5 °C–37.5 °C) using a heating lamp. Four coagulation and hemorheological parameters were detected. The rats were anaesthetized with 3% chloral hydrate. Blood was collected from the abdominal aorta and mixed with 3.2% sodium citrate at a v/v ratio of 1:9. Then, the blood was centrifuged at 3000 rpm for 10 min. The obtained plasma was analysed using an automatic coagulation apparatus (CA1500). The whole blood viscosity, plasma viscosity, erythrocyte aggregation index and Carson viscosity were measured using an automated blood rheometer. The CBF was measured at 1, 6, 12 and 24 h after MCAO/R by Laser speckle contrast imaging (LSCI) (PeriScan PSI System, Perimed, Stockholm, Sweden).

### Drug treatment

Edaravone (EDA), a free radical scavenger (Biomedical Engineering Centre, Hebei Medical University, China, Number: H20090353), was used as a positive control. The rats were randomly divided into six groups: (1) Sham group: sham rats received 0.9% NaCl intraperitoneally (*i.p.*); (2) MCAO group: MCAO rats received 0.9% NaCl *i.p.*; (3) MCAO + EDA group: MCAO rats received EDA (3 mg/kg, *i.p.*); (4) MCAO + wasp venom group (0.05, 0.2 and 0.6 mg/kg, *i.p.*). After MCAO for 1.5 h, the rats were given normal saline or wasp venom at 1.5, 22.5 and 46.5 h after ischaemia-reperfusion.

### Behaviour tests

#### Longa test

The rats were graded using a 5-point scale described previously (Longa et al. 1989; Frank-Cannon et al. 2009). In detail, the scoring criteria for the Longa test were set as follows: 0, without observable deficits; 1, unable to fully extend the left forepaw; 2, circling continuously to the left; 3, failure to the left; and 4, death or unable to move spontaneously.

#### Grip test

The grip strength (The Chatillon^®^ DFE Digital Force Gauge DFX-050) was used to evaluate the effects of wasp venom on the muscular incoordination of rats. The forelimbs of the rats were placed on the test grid, and the rats were gently pulled after grasping them. The force value when the claws of the rats were recorded was the grip value.

#### Rotarod test

The rats were placed on Rota Rod Treadmills (Harvard Apparatus, LE8205) for adaptive training three days before the MCAO/R and the rotating rod was started. The speed was set to accelerate to 40 rpm in 5 min and the rat drop time was recorded as an indicator. The tests were performed at 24 and 48 h after MCAO/R.

### Histopathological assessment

#### TTC staining

2,3,5-Triphenyltetrazolium chloride (TTC) staining was used to assess cerebral infarction. The TTC solution was prepared in phosphate-buffered solution (PBS, pH = 7.4) in the dark at 37 °C immediately before use. For this assessment, the brain was rapidly isolated and sliced into 2-mm-thick coronal sections. Subsequently, the brain slices were stained with 0.2% TTC solution at 37 °C for 15 min and then fixed with 4% paraformaldehyde (PFA) for 24 h. The infarct volume was analysed using Image-Pro Plus version 6.0 image analysis software.

#### Hematoxylin-eosin (H&E) and Nissl staining

The rats were deeply anaesthetized after 48 h of cerebral reperfusion (I/R). Their brains were collected and fixed with 4% PFA for 12–18 h, followed by dehydrating using an automatic dehydrator (Leica ASP-300S, Germany) and embedding in paraffin (Biological tissue embedding machine, Xiaogan Hongye Medical Instrument Co., Ltd., model: BM-VIII). Then, each brain sample was coronally cut (4 µm) at the hippocampus (bregma: −3.00 to −3.80 mm) using a rotatory microtome (Leica RM2245, Germany). The sections were subsequently stained with H&E or Nissl stains. Each section was observed at ×10 or ×40 magnification.

#### TUNEL staining

As mentioned above, these sections were randomly selected for TdT-mediated dUTP nick-end labelling (TUNEL) staining (TUNEL kit, Merck ApopTag) to identify apoptotic cells. Three confocal microscope fields (Leica SP8, Germany) were randomly selected from the ischaemic marginal region to count the TUNEL-positive cells. For the evaluation of the TUNEL staining results, the following equation was used to calculate the apoptosis rate:

Apoptosis rate=positive cells/total number of cells per field × 100%

### Blood–brain barrier (BBB) permeability

In order to evaluate whether the blood–brain barrier (BBB) integrity was involved in the neuroprotective effects of wasp venom, the contents of Evans blue (EB), a blood–brain permeability tracer, were detected in the ischaemic cortex. Briefly, 2% EB was intravenously administered and circulated *in vivo* for at least 90 min. Then, the brains were removed from the rats and sliced into 2-mm-thick coronal sections and photographed. Then, the whole brain was collected and weighed. The contents of EB were determined as previously described (O-Uchi and Tanaka 1988).

### Transmission electron microscopy (TEM)

The frontal cortices were isolated from the rats with stroke, fixed with 2% glutaraldehyde and 4% PFA in 0.1-M sodium cacodylate (pH = 7.4), treated with 10% gelatine solution in sodium cacodylate buffer, and incubated with 2% osmium tetroxide. Then, they were sliced into 40 nm thick sections using an EM TP ultramicrotome (Leica, Germany), and placed within grids stained with a 1:1 mix of 3% uranyl acetate and 50% acetone for 30–50 s. The grids were observed under a JEM 1400 transmission electron microscope (JEOL, Tokyo, Japan) at ×1200 for low magnification and ×12,000 for high magnification (unless otherwise noted) using Gatan Microscopy Suite software (Gatan, Pleasanton, CA).

### Cytokine enzyme-linked immune sorbent assays (ELISAs)

After 48 h of MCAO/R, the brains were collected and homogenized in PBS (pH = 7.4, 5% weight/volume); then, the resultant homogenates were centrifuged at 3000 rpm for 5 min at 4 °C. The supernatants were obtained and used for the ELISA. The interleukin-1 beta (IL-1 β, 147425023), tumour necrosis factor-alpha (TNF-α, 147881045) and interleukin-6 (IL-6, 146379036) were purchased from Thermo Fisher Scientific, Waltham, MA. According to the manufacturer’s specifications, the levels of these cytokines were measured by ELISA kits.

### Quantitative immunoblotting

After 48 h of MCAO/R, ischaemic hemisphere in brains was collected and disrupted using RIPA buffer (high) (Shandong Sparkjade Biotechnology Co., Ltd.). Quantitative immunoblotting was performed as previously described (Hu et al. 2021). Anti-β-tubulin (Millipore, Burlington, MA) was used as an internal loading control. The images of blots were captured using an Azure C300 with secondary antibodies. The images were captured using Image Lab, and the signal intensities were normalized to loading controls. The antibodies and their concentrations are as following: (antibody, company, catalog number, dilution): Anti-Claudin-5, Millipore, ABT45, 1:500; and Anti-Aquaporin-4 (AQP4), Millipore, ABN411, 1:500.

### Immunohistochemistry

Immunohistochemistry (IHC) was used to detect the expression of fibroblast growth factor (FGF) and vascular endothelial growth factor (VEGF) in the infarct area. Briefly, the antigen was retrieved by microwave-heating the tissue in a 10 mM sodium citrate buffer at pH = 6.0. These sections were then blocked for 1 h with a blocking solution (0.1% Triton-X, 10% normal goat serum in 1× PBS) at room temperature (RT). After that, the antibodies FGF-2 (1:100, 05-118, Millipore), VEGF (1:100, ABS82, Millipore) and IBA-1 (1:400, 019-19741, WAKO, Japan) were added to these tissue samples, respectively, and placed overnight at 4 °C. The samples were then incubated using biotin-labelled secondary antibodies at RT for 30 min. The HRP-labelled SP working medium was added and incubated at RT for 30 min.

### Statistical analysis

**Figure 1.**
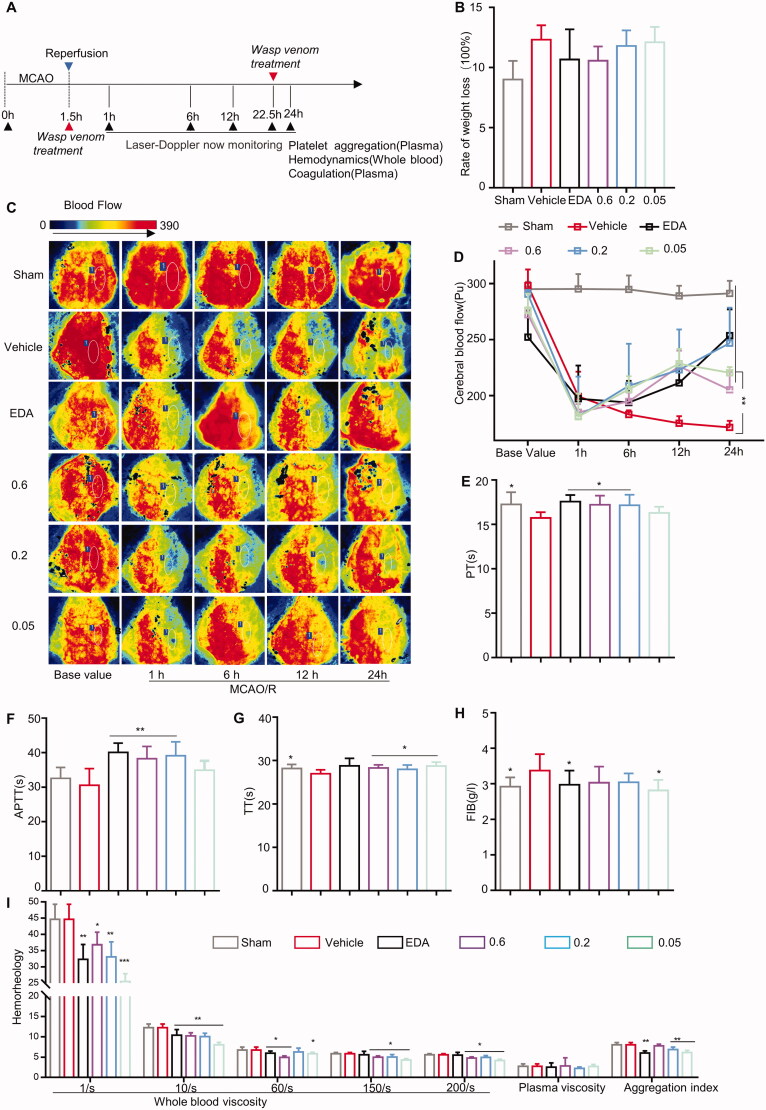
Wasp venom improved the blood flow after middle cerebral artery occlusion (MCAO) in rats. (A) The administration of wasp venom schedule and the detection of cortical blood flow (CBF), hemodynamic parameters, and four coagulation parameters are illustrated schematically. (B) The rate of weight loss. (C) Laser speckle contrast imaging (LSCI) was displayed after MCAO/R at baseline, 0, 1, 6, 12 and 24 h in rats. (D) CBF quantification with Laser Perfusion Imager Review V5.0 software and expression as ipsilateral mean perfusion in two ROIs. (E–H) Four coagulation parameters were analysed using an automatic coagulation apparatus (CA1500). (I) Hemorheological parameters displayed respectively, with whole blood viscosity at different shear rates of 1/s, 10/s, 40/s, 150/s and 200/s, plasma viscosity, and erythrocyte aggregation index. Statistical analyses were performed using Bonferroni’s multiple comparisons tests (*p<.05, **p<.01 versus the vehicle-treated group). All the data are presented as means ± SEM (*n* = 6–8 per group).

**Figure 2.**
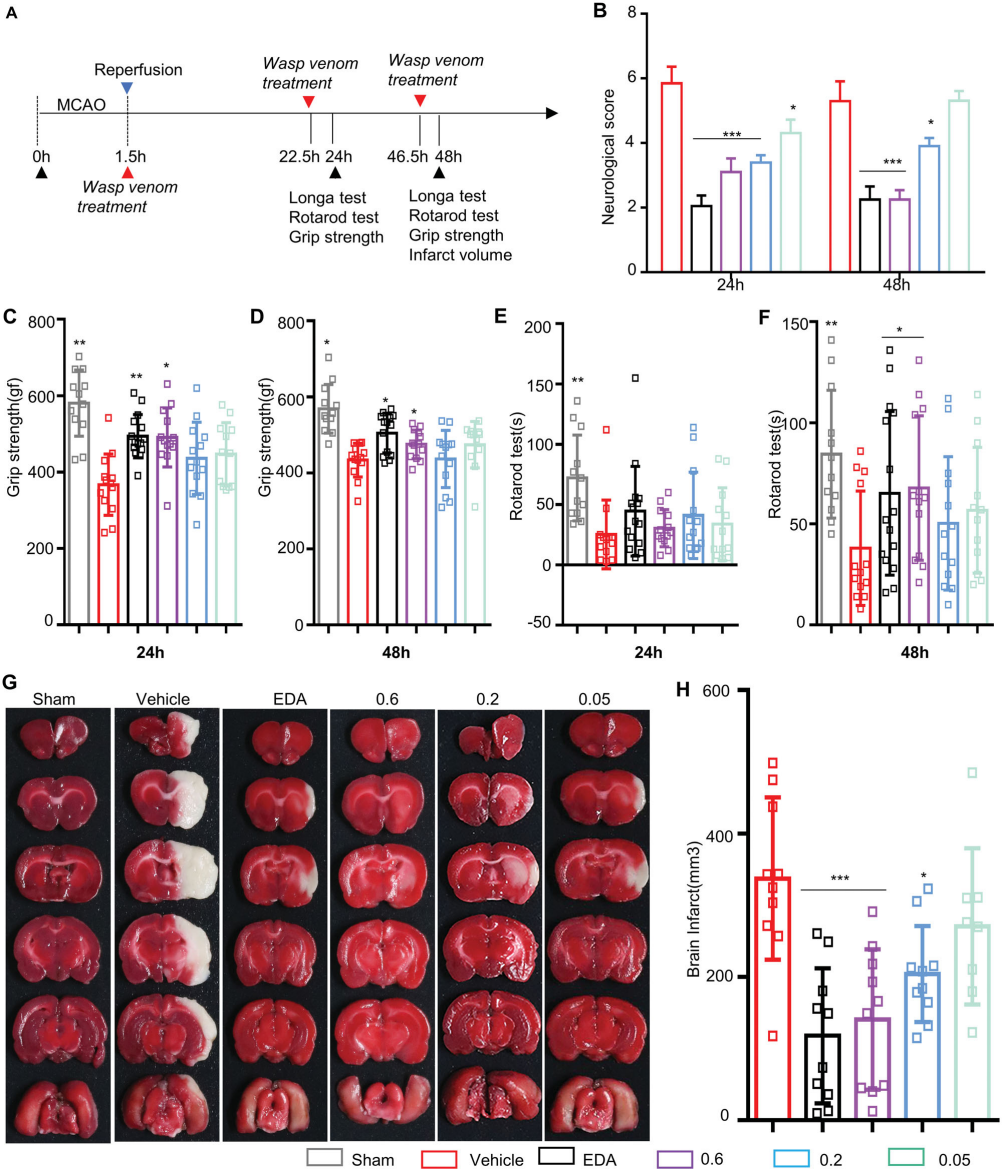
Effects of wasp venom on sensorimotor functions and brain infarct volume. (A) The administration of wasp venom schedule and behavioural assessment timeline are illustrated schematically. The wasp venom treatment improved sensorimotor recovery as evaluated by the Longa test (B), the grip test (C, D), and the Rotarod test (E, F) 24 and 48 h after MCAO. The performance of the Rotarod test was expressed as the time spent on the rotating rod before falling off, and the Grip test was expressed as the score of pull-up time for each mouse. (G) Representative triphenyltetrazolium chloride (TTC) staining images of the coronal sections 24 h after reperfusion. (H) The wasp venom treatment reduced the cerebral infarction volume as assessed by TTC staining 48 h after MCAO/R. The statistical analyses were performed using Bonferroni’s multiple comparisons tests (*p<.05, **p<.01, ***p<.001 *versus* the vehicle-treated group). Data were expressed as means ± SEM (*n* = 8-15).

Statistical analysis was conducted using Graph Pad Prism version 8 software (La Jolla, CA). Four coagulation (Figure 1(E–H)), haemorheological parameters (Figure 1(I)) and Behaviour test (Figure 2(B–F)) were analysed using Kruskal–Wallis test, followed by Dunn’s multiple comparisons test. Cerebral blood flow was analysed with two-factor repeated measures analysis of variance, multiple comparison analysis (Figure 1(D)). Other detected data basically fit the normal distribution with one-way ANOVA statistical analysis. Statistical difference was established at *p*<0.05.

## Results

### Wasp venom promotes CBF recovery

Composition of wasp venom has been described as previously (Zhou et al. 2019). Here, the surgery and drug treatments were carried out as shown in Figure 1(A). The transient MCAO/R injury, mimicking the cerebral ischaemic stroke and reperfusion in rodents, has been widely used to evaluate the effects of the drug on AIS (Fisher et al. 2009; Liesz et al. 2009; Shichita et al. 2009, 2012). The CBF levels were measured 0, 1, 6, 12 and 24 h after MCAO/R in rats to determine the potential effects of wasp venom on stroke. The baseline of each rat was set to 100% before MCAO induction. A similar rate of weight loss was found in each group of rats (Figure 1(B)). LSCI images were captured after MCAO/R in rats (Figure 1(C)). Compared to the sham group, a decrease in CBF was observed in the vehicle group (at 1, 6, 12 and 24 h, *p*<0.01) (Figure 1(D)). The therapy with wasp venom (0.05, 0.2 and 0.6 mg/kg) promoted the CBF recovery after MCAO/R (at 12 h, *p*<0.05; at 24 h, *p*<0.01) (Figure 1(C)). Collectively, these data suggested that the MCAO-induced shortage of blood supply to the brain could be partially restored to the normal level by treating with wasp venom.

According to the evidence, the factors of endothelium dysfunction, activation of coagulation factor (Kleinschnitz et al. 2007; De Meyer et al. 2012, 2016), and decreased fibrinolysis function (Docagne et al. 2015) play an essential role in the pathogenesis of stroke. Compared to the sham group, abnormal coagulation was observed in the vehicle-treated group (*p* < 0.05) as follows: the prothrombin time (PT), activated partial thromboplastin time (APTT) and thrombin time (TT) decreased; fibrinogen (FIB) increased (Figure 1(E–H)). Compared to the vehicle group, the wasp venom could increase the APTT (0.2 and 0.6 mg/kg, *p*<0.01), PT (0.2 and 0.6 mg/kg, *p*<0.05), and TT (0.02 mg/kg, *p*<0.05), suggesting a noticeable effect of wasp venom on the endogenous and exogenous coagulation pathways.

The increased whole blood viscosity correlated with the decreased CBF in the ranges measured in cerebral ischaemic stroke patients, and the changes in viscosity might have an important effect on the CBF in regions where the flow is low (Grotta et al. 1982; Li et al. 2015). As shown in Figure 1(I), hemodynamic parameters were detected. No difference in the whole blood viscosity was observed at different shear rates between the vehicle-treated and sham group rats (*p*>.05) (Figure 1(I)). Compared to the vehicle group, the wasp venom treatment decreased the blood viscosity at 1/s, 10/s (*p*<0.01), 150/s and 200/s (*p*<0.05) (Figure 1(I)). The wasp venom could reduce the whole blood viscosity at 40/s (*p*<0.05) (Figure 1(I)). Compared to the sham group, the plasma viscosity and erythrocyte aggregation index insignificantly decreased in the vehicle group. Compared to the vehicle group, no significant difference was observed in the plasma viscosity by wasp venom (Figure 1(I)). However, the wasp venom treatment reduced the red blood cell aggregation index (*p*<0.01). These results suggested that the wasp venom could decrease blood viscosity.

### Wasp venom alleviates neurological impairment and cerebral infarction

For the MCAO/R model, the drug treatments and behavioural tests were performed, as shown in Figure 2(A). Except for the sham group (*n* = 12), 120 remaining rats underwent MCAO surgery. In order to ensure the MCAO-induced cerebral ischaemia in rats, LSCI was used to monitor the CBF. The results showed that the CBF dropped by 25% of the initial blood flow. During the surgery, blood gases, body temperature and blood pressure of all the rats were within the normal range, with no differences between the groups (Table 1). In order to investigate the neurological function of rats following MCAO/R, the effects of wasp venom on sensorimotor were investigated by a series of behavioural tests. As shown in the Longa test, the vehicle-treated rats significantly exacerbated deficits in the mobility of the left limbs compared to the sham group (Figure 2(B)). However, the administration of wasp venom improved neurological deficits (at 24 h, 0.2 and 0.6 mg/kg, *p*<.001, 0.05 mg/kg, *p*<0.05; at 48 h, 0.2 and 0.6 mg/kg, *p*<.001) compared to the vehicle-treated rats (Figure 2(B)). There were no significant differences between the wasp venom groups (0.2 and 0.6 mg/kg) and EDA (3 mg/kg).

**Table 1.** Physiological parameters at post-wasp venom treated in stroke rats.

Groups	Variables				
	PaO_2_ (mmHg)	BP (mmHg)	PaCO_2_ (mmHg)	Temperature (°C)	pH value
Sham	88.69 ± 3.84	98.87 ± 3.91	41.40 ± 2.71	35.72 ± 0.33	7.39 ± 0.02
Vehicle	90.06 ± 4.08	96.54 ± 2.73	40.67 ± 1.97	35.89 ± 0.30	7.39 ± 0.02
EDA	87.82 ± 4.31	98.02 ± 3.52	40.79 ± 1.60	35.73 ± 0.24	7.32 ± 0.02
0.6	87.45 ± 3.22	96.13 ± 2.98	41.36 ± 1.78	35.81 ± 0.29	7.39 ± 0.01
0.2	88.55 ± 3.05	96.35 ± 3.42	41.54 ± 2.16	35.86 ± 0.28	7.39 ± 0.02
0.05	87.58 ± 2.67	97.03 ± 4.66	41.55 ± 2.06	35.85 ± 0.34	7.38 ± 0.02

In the grip test, the MCAO/R revealed persistent circling movements with severe paw flection and curved posture towards the paretic side. Significantly impaired motor coordination and weak grip strength were also observed in the vehicle group compared to the sham group (at 24 h, *p*<0.01; at 48 h, *p*<0.05) (Figure 2(C,D)). However, a marked improvement in the grip test was observed in the wasp venom groups (at 24 h, 0.6 mg/kg, *p*<0.05) compared to the vehicle-treated group (Figure 2(C)). The wasp venom groups showed no significant grip strength and motor coordination at 48 h (Figure 2(D)).

Compared to the sham group, a significant difference was observed in the vehicle-treated group in the motor performance similar to the Rotarod test (at 24 h, *p*<0.01) (Figure 2(E)). In the wasp venom groups (0.05, 0.2 and 0.6 mg/kg), the differences observed in the motor performance at 24 h were insignificant (Figure 2(E)); however, there was an improvement in the motor performance at 48 h (Figure 2(F)).

In order to study the effects of wasp venom on infarct volume, the damaged area of the rats sacrificed at 48 h after MCAO/R was analysed. There was no infarct area in the sham group, while the MCAO/R groups showed infarct areas, including the striatum, hippocampus, cortex and caudate nucleus (Figure 2(G–H)). The wasp venom decreased the infarct volume (0.6 mg/kg, *p*<0.05; 0.2 mg/kg, *p*<0.01). Compared to the EDA group, no significant difference was observed between the wasp venom treatment groups (*p*>.05). Taken together, these findings indicated that the wasp venom therapy in rats alleviated the neurological impairment and reduced the infarct volume, representing a potential therapeutic target for brain injuries.

### Wasp venom maintains the integrity of BBB

**Figure 3.**
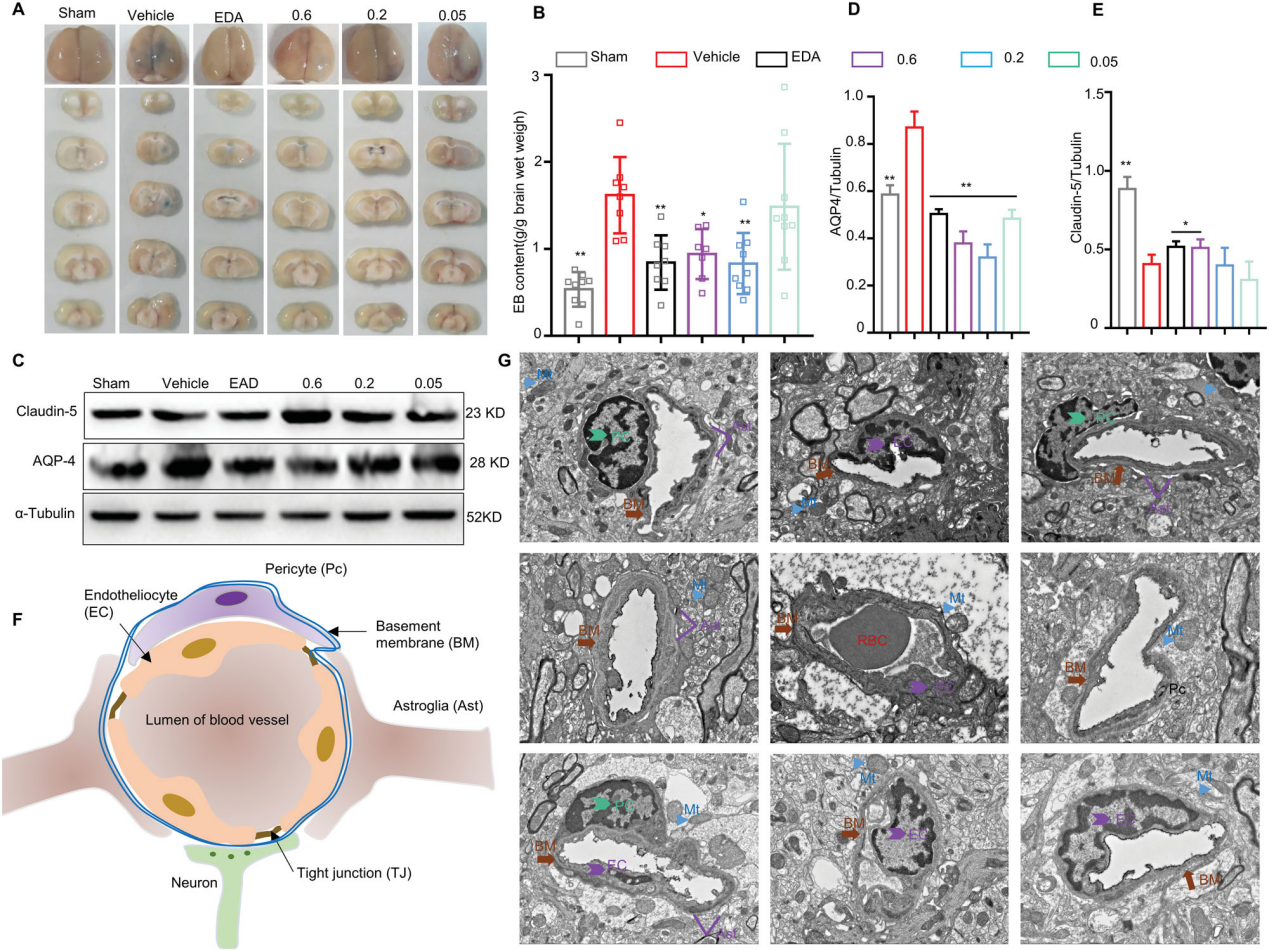
Protective effect of wasp venom on the blood–brain barrier (BBB). (A) Brain sections were stained with EB. (B) EB content in damaged (right) hemispheres was quantified. (C) Changes in the expression of aquaporin-4 (AQP-4) and claudin-5 were detected using immunoblotting and quantified using Image Lab (D,E). (F) A schematic representation of BBB. The BBB consisted of mainly endothelial cells (EC), pericytes (PC), astrocytes (Ast), neurons, tight junction (TJ) and basement membranes (BM). The main components of the BBB included cerebral microvascular ECs that were joined by TJ, restricting the exogenous molecules in the brain. (G) The structure of BBB was observed using a transmission electron microscope. In the sham group (G1–3), the capillary morphology was regular; EC and TJ were complete; the thickness of BM was uniform and continuously around the outside of EC; the structure of Mt was clear. When the BBB was destroyed (G4–6), the BM and TJ were largely dissolved and shed; the Ast had extreme edoema; there were large electronic blank areas, and the Mt was loose or vacuolar, which were relieved by the wasp venom (0.6 mg/kg) treatment (G7–9). Mitochondria, Mt. The marks with different symbols indicate the constituent cells or matrix of BBB. Scale bars: 2 µm. Data are expressed as the means ± SD (*n* = 7–9). Values were analysed using one-way analysis of variance (ANOVA) with Tukey’s multiple comparisons tests (*p<.05, **p<.01, ***p<.001 *versus* the vehicle treatment group).

The destruction of BBB during cerebral I/R is a significant pathological change that induces cerebral oedema and cerebral infarction (Warach and Latour 2004; Simard et al. 2007). The permeability of BBB was assessed using EB staining and TEM. The stained area of the right hemisphere in the vehicle group increased (Figure 3(A)). As shown in Figure 3(B), the vehicle-treated group exhibited a marked increase in the EB extravasation, while the wasp venom (0.2 mg/kg, *p*<0.01; 0.6 mg/kg, *p*<0.05) and EDA groups (*p*<0.01) reversed the effects of MCAO-induction on the BBB permeability. The pathological alterations of tight junctions (Abbott et al. 2006), especially the AQP-4 (Hirt et al. 2017) and claudin-5 (Nitta et al. 2003), affected the BBB function, especially the barrier permeability, during an ischaemic stroke. Herein, the changes in the claudin-5 and AQP-4 expression were investigated (Figure 3(C)). Importantly, apparent down-regulation of AQP-4 and up-regulation of claudin-5 were detected in the wasp venom groups (Figure 3(D,E)). The BBB function depends on the integrity of its components, especially the microvascular endothelial cells and astrocytes involved in its formation (Figure 3(F)). The wasp venom could maintain the integrity of BBB (Figure 3(G)). These results collectively demonstrated that the wasp venom protected cerebral ischaemic injury by maintaining the BBB permeability.

### Wasp venom suppresses inflammatory response and apoptosis

**Figure 4.**
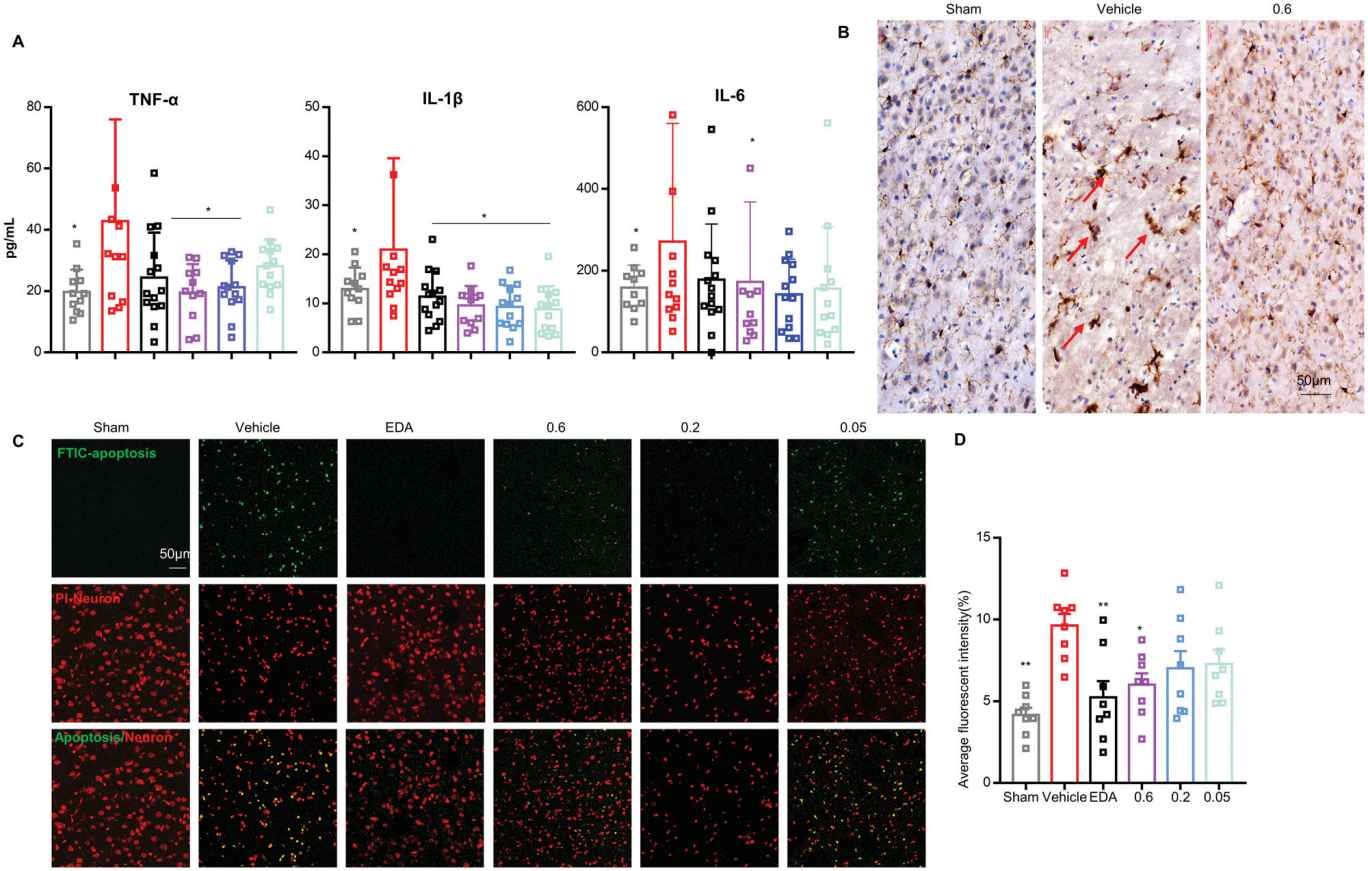
Effect of wasp venom on the neuroinflammation and apoptosis after MCAO/R in rats. (A) The levels of the pro-inflammatory cytokines (interleukin (IL)-1beta (1β), tumour necrosis factor-alpha (TNF-α), and IL-6) in the ischaemic penumbra of rats at 48 h were detected by ELISA. (B) IHC was performed to observe the morphology of microglia labelled with anti-Iba1 antibody. (C) Representative confocal images of TUNEL staining in the cerebral cortex. (D) Quantitative analysis of the number of TUNEL-positive neurons in each group. Data are expressed as means ± SD. (*n* = 8–15 for each group, *p<.05, **p<.01 *versus* the vehicle treatment group; scale bars: 50 µm).

Acute inflammation plays a prominent role in the pathogenic progression of stroke. The wasp venom has been investigated as a neuropeptide for various neurological disorders. A previous study reported that the venom from *Habrobracon hebetor* Say (Hymenoptera: Braconidae) could dose-dependently abolish the production of nitric oxide (NO) and inhibit the levels of pro-inflammatory mediators (Saba et al. 2017). The production of pro-inflammatory IL-1 β, TNF-α and IL-6 in the ischaemic hemisphere of rats was determined at 48 h to investigate the anti-inflammatory effects of wasp venom on MCAO/R. In this study, the pro-inflammatory cytokines, including TNF-α, IL-1β and IL-6, increased in vehicle-treated rats compared to those in the sham group (*p*<0.05) (Figure 4(A)). In comparison to the vehicle group, the wasp venom treatment significantly decreased TNF-α (0.2 and 0.6 mg/kg, *p*<0.05), IL-1β (0.05, 0.2 and 0.6 mg/kg, *p*<0.05), and IL-6 (0.6 mg/kg, *p*<0.05) (Figure 4(A)). There were insignificant differences between the wasp venom group (0.6 mg/kg) and the EDA group. Meanwhile, the IHC was performed to observe the activation of microglia by IBA-1. A marked elevation in the IBA-1 expression was observed in the cerebral cortex of rats after cerebral I/R, which was greatly inhibited by the administration of wasp venom (0.6 mg/kg) (Figure 4(B)). The microglia in the sham group were in a resting state, characterized by the small cell bodies and elongated branches (Nayak et al. 2014), while they became larger with shorter and thicker axons, which was considered the activated state (Perry et al. 2010) in the vehicle-treated group. The morphology of microglia in the wasp venom group was in-between that of the previous groups. These results showed that the wasp venom could inhibit the neuro-inflammatory response and provide a novel approach for treating ischaemic stroke.

**Figure 5.**
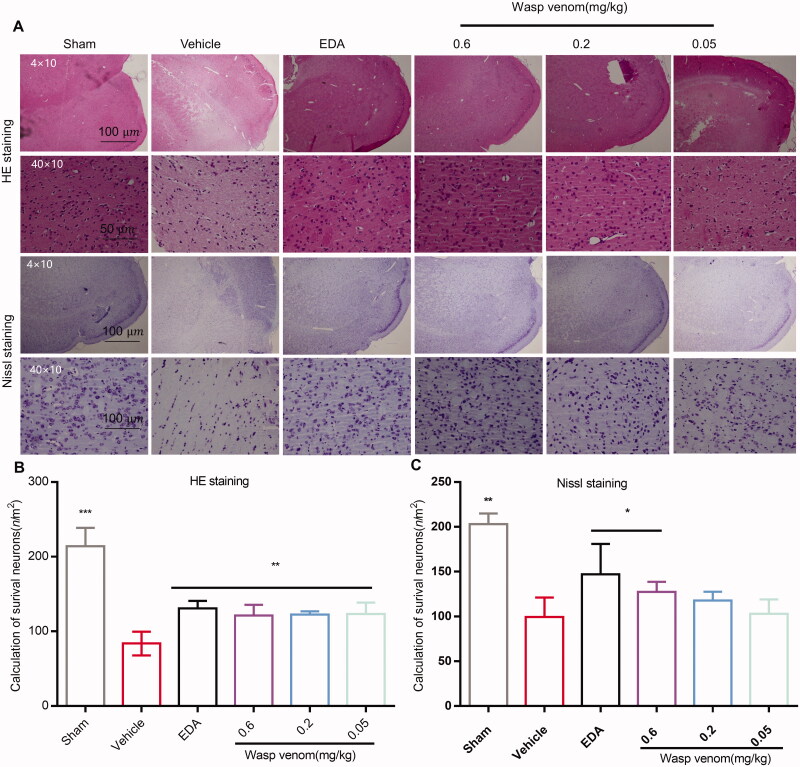
Effects of wasp venom on the histopathological changes in the cortex of MCAO/R rats. (A) HE staining and Nissl staining in coronal sections from the cerebral cortex are displayed. The vehicle-treated group showed a typical appearance of neuron necrosis and inflammatory cell infiltration; the cell types were difficult to identify. In contrast, the rats treated with wasp venom showed less extensive damage. (B,C) The quantification of the number of survived neurons in each group by H&E staining and Nissl staining (Scale bars: 50 µm; magnification: ×4 or ×40). Data are expressed as means ± SD (*n* = 4–6 for each group, *p<.05, **p<.01 *versus* the vehicle treatment group).

The TUNEL staining results are shown in Figure 4(C,D). Extensive TUNEL-positive cells were observed in the vehicle-treated group. In contrast, the number of TUNEL-positive cells in the wasp venom-treated (0.6 mg/kg) group was significantly lower compared to the vehicle-treated group (*p*<0.05) (Figure 4(D)). This finding was consistent with the H&E and Nissl staining (Figure 5(A–C)). These observations indicated that the wasp venom decreased the ischaemia-induced apoptosis.

### Wasp venom regulates angiogenesis factors

**Figure 6.**
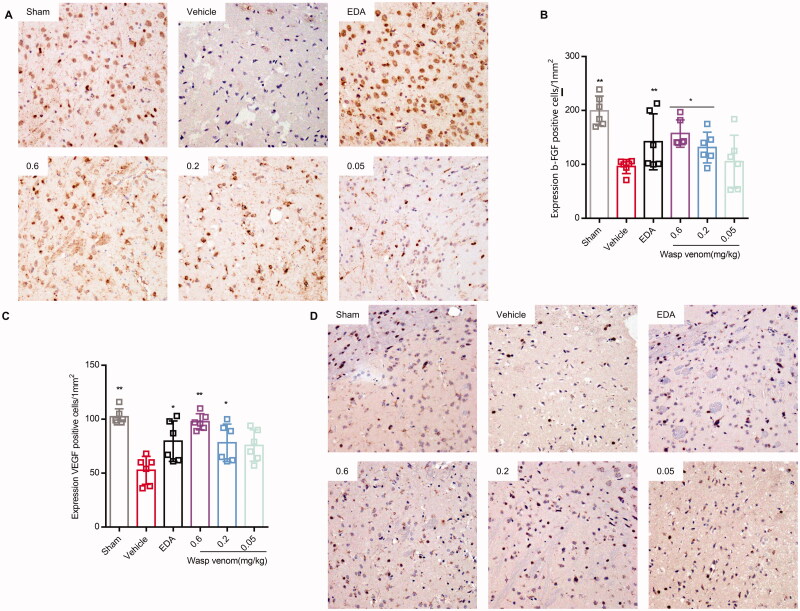
Effects of wasp venom on the expression levels of angiogenesis factors after MCAO/R induction. The representative immunohistochemistry (IHC) for (A) fibre cell growth factor (FGF) and (D) vascular endothelial growth factor (VEGF) was performed (Scale bar: 50 μm). The number of (B) b-FGF and (C) VEG-positive cells in the penumbra region were normalized and counted as cells/mm2. Three micrographs with different magnifications for the experimental groups are shown (n = 5-6 per group). Data are presented as means ± SD (*p<.05; **p<.01 *versus* vehicle group by one-way ANOVA with Bonferroni’s multiple comparisons test).

Recent studies have demonstrated a therapeutic effect of wasp venom on the neurogenesis and angiogenesis factors of stroke (Krupinski et al. 1994; Zhang and Chopp 2015). The therapy increased angiogenesis, which has also been proved to be beneficial in animal stroke models. Lastly, to evaluate the effects of wasp venom on angiogenesis factors after ischaemia-reperfusion, the coronal sections were collected, and IHC was performed for FGF and VEGF markers (Figure 6(A–D)). As shown in Figure 6(A,B), poor b-FGF positive cells in the cerebral cortex region could be seen in the vehicle group (*p*<0.01) (Figure 6(B)), while that in the penumbra zone of ischaemia increased in wasp venom (0.2 and 0.6 mg/kg, *p*<0.05) or EDA (*p*<0.01) treatment groups compared to the vehicle group. Subsequently, the VEGF-positive cells in the sham group were largely expressed, while in the vehicle-treated group, the VEGF-stained cells significantly decreased *(p*<0.01). Importantly, the wasp venom treatment (0.2 mg/kg, *p*<0.05; 0.6 mg/kg, *p*<0.01) prevented the down-regulation of VEGF-positive cells in the cerebral cortex region *versus* vehicle-treated rats (Figure 6(D)). Therefore, the results indicated that wasp venom regulates angiogenesis factors (VEGF, b-FGF) after MCAO/R in rats at 48 h.

## Discussion

Previous studies have shown that bee venom could normalize all neuro-inflammatory (Moon et al. 2007) and apoptotic markers and restore the neurochemical functions of the brain, which might prove a promising neuroprotective therapy for PD (Chung et al. 2012; Khalil et al. 2015) or ALS (Yang et al. 2010; Cai et al. 2015). Other toxins such as snake venom, spider venom, scorpion venom, etc., mastoparan M extracted from wasp venom is also a kind of neurotoxin and cell penetrating peptide (Rádis-Baptista 2021) increasing drug concentration in the brain (Chen Y and Liu 2012). The wasp venom or its compounds has higher cytotoxicity (Jones and Howl 2012). Compared to bee venom, few pharmacological investigations have been undertaken for wasp venom. In this study, low-dosage of wasp venom (*i.p.*) was used to treat stroke in rats. Doses of 0.05, 0.2 and 0.6 mg/kg were selected as evidenced by our previous study (Gao et al. 2020) and other reports (Kim et al. 2016; Badawi et al. 2020), mainly based on the safety and effectiveness of wasp venom. In our earlier investigation, we identified different compounds in wasp venom. Currently, in according to the Stroke Therapy Academic Industry Roundtable (STAIR) recommendations (Fisher et al. 2009), we investigated a dose-response experiment of wasp venom treatment on stroke outcomes by MCAO(/R in rats. The MCAO-induced cerebral I/R injury has been the most frequently used because its pathological process is very similar to clinical AIS. A successful stroke model was confirmed by LSCI (Ren et al. 2020). For the MCAO model, the infarct size implicated in caudate-putamen, striatum, and cortex (Tobin et al. 2014). Our results showed that the wasp venom improved brain damage, including the acceleration of CBF, recovery of neurological impairment, reduction of cortex infarct volume, prevention of apoptosis, and inflammatory response. The MCAO-induced AQP-4 aggravated a declination of CBF, leading to a different evolution of the brain injury. However, a significant increase in CBF was detected in the wasp venom group after the stroke at 12 or 24 h. Accumulating evidence shows that the adhesion of platelets to brain microvascular leukocytes and endothelial cells is caused by the absence of reflow, which could further damage the tissues after the activation of platelets by I/R reperfusion (Pan et al. 2007). Our findings also demonstrated that the and clotting time lengthened by the administration of wasp venom, suggesting that the protective effects of wasp venom were also implicated in blood viscosity and coagulation. Therefore, the wasp venom was suggested to promote the recovery of CBF in the ischaemic penumbra.

In the model of the MCAO/R rat, it was also reported that the wasp venom could effectively improve the recovery of sensorimotor and reduce the infarct volume and permeability of BBB. In patients, the ischaemic stroke arose from complex pathophysiological mechanisms, with different clinical manifestations (Bogousslavsky 1991; Song 2011). The presence of the standardized scores for sensorimotor in rats provided an advantage in functional assessment (Liu and McCullough 2011). The reason is that rats and humans have similar cerebral blood circulation (Chen ST et al. 1986). We demonstrated that the wasp venom improved the sensorimotor recovery in stroke by the Longa test, Grip test, and Rotating rod test. Additionally, the positive effect of wasp venom on the potential mechanism of function recovery was also investigated.

The development of cerebral I/R injury in rats showed that the treatment with wasp venom ameliorated histological damages. The TUNEL staining also demonstrated the alleviation of cell apoptosis. It should be noted that the anti-apoptotic activities of wasp venom might not be the only factors accounting for the wasp venom-mediated neuroprotection. The wasp venom is a rich mixture of peptides, such as mastoparan, pompilidotoxins, AvTx-7, wasp kinin, biologically active amines and non-peptide components. Interestingly, the wasp kinin was the first neurotoxin component isolated from the wasp venom. This small peptide plays an important role in controlling blood pressure, kidney function, heart function and inflammation in rodents (Moreau et al. 2005). Moreover, the treatment with wasp kinin significantly attenuated the ischaemia-induced neuronal death, prevented the caspase-3 activation, and suppressed the release of superoxide dismutase (SOD) (Danielisova et al. 2009). Similarly, our study also indicated wasp kinin purification and identification, suggesting that wasp venom could inhibit activated microglia and anti-neuroinflammation.

Furthermore, it has been demonstrated that neurological impairment in stroke is mediated by inflammatory reactions and pro-inflammatory cytokines (Jin et al. 2010; Herz et al. 2014). The wasp venom significantly reduced the release of pro-inflammatory mediators, including TNF-α, IL-1β and IL-6, suggesting that it could relieve neuro-inflammatory responses. Importantly, the wasp venom inhibited the activation of microglia and apoptosis.

When cerebral ischaemia occurs, the brain tissues immediately initiate endogenous repair mechanisms, such as angiogenesis and synaptic remodelling. Angiogenesis is the process of growing new blood vessels from the existing ones, enabling the rapid establishment of new vascular networks to supply ischaemic tissue in a short period (Zhang ZG et al. 2002; Arai et al. 2009). Mechanistic studies have revealed that the neuroprotective effects of wasp venom were related to the upregulation of VEGF and b-FGF. These results potentially explained the preventive effects of wasp venom against ischaemic brain injury in clinical practice. Therefore, the wasp venom exhibits a positive impact on the angiogenesis and prognosis of stroke patients.

## Limitations of study

First, wasp venom was administrated at the start of reperfusion. Pharmacological effects of wasp venom, such as recovery of CBF, decreased blood viscosity and lengthened clotting time, could also be due to reperfusion. Whether wasp venom is effective against permanent cerebral ischaemia should be profoundly researched and reconsidered. This was also a design limitation of the experiment. Clinically, cerebral I/R injury is treated by neuroprotective drugs, we were interested in neuroprotective effects of wasp venom for successful reperfusion in this article. Second, EDA, a free-radical scavenger, and in 2001 is a neuroprotective agent that have been approved in Japan and China for the treatment of patients with AIS. In clinical studies, EDA improved the core neurologic deficits, activities of daily living, and functional outcome of stroke patients (Enomoto et al. 2019; Kobayashi et al. 2019). In regard to CBF and fibrinolytic activity, EDA as a positive control is not ideal. Our results exhibited that it also affects blood coagulation, reduces blood viscosity and improves CBF. Although, EDA is not an anticoagulation or thrombolytic drug or anti-platelet aggregation drug. As the brain injury recovered during the development of the stroke, other parameters such as blood coagulation, blood viscosity and the CBF could return to normal. Compounds isolated from wasp venom were also called neurotoxins for the treatment of neurodegenerative diseases, such as pompilidotoxins, Mastoparan, AvTx-7, wasp kinin. Interestingly, crude venom provoked severe generalized tonic-clonic seizures, respiratory depression and death. On the other hand, in this study, low doses of denatured venom had an effect on stroke. We consider that the remaining potential applications described here are currently in earlier stages of investigation and their conversion into realistic therapeutic or biotechnological applications still need to be explored.

## Conclusions

The described results confirmed that the wasp venom inhibited neuroinflammation and apoptosis. These pharmacological effects were related to the interaction of the multiple components of the venom. In the future, we will focus on determining whether the observed actions were due to a single compound or the interaction of multiple components of venom, along with the investigation of the detailed mechanism of neuroprotection by wasp venom.

## References

[CIT0001] Abbott NJ, Rönnbäck L, Hansson E. 2006. Astrocyte-endothelial interactions at the blood-brain barrier. Nat Rev Neurosci. 7(1):41–53. 16371949 10.1038/nrn1824

[CIT0002] Amara SG. 1992. Neurotransmitter transporters. A tale of two families. Nature. 360(6403):420–421. 1333057 10.1038/360420d0

[CIT0003] Arai K, Jin G, Navaratna D, Lo EH. 2009. Brain angiogenesis in developmental and pathological processes: neurovascular injury and angiogenic recovery after stroke. FEBS J. 276(17):4644–4652. 19664070 10.1111/j.1742-4658.2009.07176.x PMC3712842

[CIT0004] Araki N, Greenberg JH, Sladky JT, Uematsu D, Karp A, Reivich M. 1992. The effect of hyperglycemia on intracellular calcium in stroke. J Cereb Blood Flow Metab. 12(3):469–476. 1569140 10.1038/jcbfm.1992.64

[CIT0005] Badawi HM, Abdelsalam RM, Abdel-Salam OM, Youness ER, Shaffie NM, Eldenshary EDS. 2020. Bee venom attenuates neurodegeneration and motor impairment and modulates the response to L-dopa or rasagiline in a mice model of Parkinson's disease. Iran J Basic Med Sci. 23(12):1628–1638. 33489038 10.22038/ijbms.2020.46469.10731 PMC7811814

[CIT0006] Baron JC. 1999. Mapping the ischaemic penumbra with PET: implications for acute stroke treatment. Cerebrovasc Dis. 9(4):193–201. 10393405 10.1159/000015955

[CIT0007] Bogousslavsky J. 1991. Topographic patterns of cerebral infarcts. Cerebrovasc Dis. 1(1):61–68.

[CIT0008] Cai M, Choi SM, Yang EJ. 2015. The effects of bee venom acupuncture on the central nervous system and muscle in an animal hSOD1G93A mutant. Toxins (Basel). 7(3):846–858. 25781653 10.3390/toxins7030846 PMC4379529

[CIT0009] Chen ST, Hsu CY, Hogan EL, Maricq H, Balentine JD. 1986. A model of focal ischemic stroke in the rat: reproducible extensive cortical infarction. Stroke. 17(4):738–743. 2943059 10.1161/01.str.17.4.738

[CIT0010] Chen Y, Liu L. 2012. Modern methods for delivery of drugs across the blood-brain barrier. Adv Drug Deliv Rev. 64(7):640–665. 22154620 10.1016/j.addr.2011.11.010

[CIT0011] Chung ES, Kim H, Lee G, Park S, Kim H, Bae H. 2012. Neuro-protective effects of bee venom by suppression of neuroinflammatory responses in a mouse model of Parkinson's disease: role of regulatory T cells. Brain Behav Immun. 26(8):1322–1330. 22974722 10.1016/j.bbi.2012.08.013

[CIT0012] Danielisova V, Gottlieb M, Nemethova M, Burda J. 2008. Effects of bradykinin postconditioning on endogenous antioxidant enzyme activity after transient forebrain ischemia in rat. Neurochem Res. 33(6):1057–1064. 18080186 10.1007/s11064-007-9550-3

[CIT0013] Danielisova V, Gottlieb M, Nemethova M, Kravcukova P, Domorakova I, Mechirova E, Burda J. 2009. Bradykinin postconditioning protects pyramidal CA1 neurons against delayed neuronal death in rat hippocampus. Cell Mol Neurobiol. 29(6–7):871–878. 19259804 10.1007/s10571-009-9369-3 PMC11505757

[CIT0014] De Meyer SF, Denorme F, Langhauser F, Geuss E, Fluri F, Kleinschnitz C. 2016. Thromboinflammation in stroke brain damage. Stroke. 47(4):1165–1172. 26786115 10.1161/STROKEAHA.115.011238

[CIT0015] De Meyer SF, Stoll G, Wagner DD, Kleinschnitz C. 2012. von Willebrand factor: an emerging target in stroke therapy. Stroke. 43(2):599–606. 22180250 10.1161/STROKEAHA.111.628867 PMC4102321

[CIT0016] De Rooij NK, Greving JP, Rinkel GJ, Frijns CJ. 2013. Early prediction of delayed cerebral ischemia after subarachnoid hemorrhage: development and validation of a practical risk chart. Stroke. 44(5):1288–1294. 23512975 10.1161/STROKEAHA.113.001125

[CIT0017] Docagne F, Parcq J, Lijnen R, Ali C, Vivien D. 2015. Understanding the functions of endogenous and exogenous tissue-type plasminogen activator during stroke. Stroke. 46(1):314–320. 25395410 10.1161/STROKEAHA.114.006698

[CIT0018] Emberson J, Lees KR, Lyden P, Blackwell L, Albers G, Bluhmki E, Brott T, Cohen G, Davis S, Donnan G, et al. 2014. Effect of treatment delay, age, and stroke severity on the effects of intravenous thrombolysis with alteplase for acute ischaemic stroke: a meta-analysis of individual patient data from randomised trials. Lancet. 384(9958):1929–1935. 25106063 10.1016/S0140-6736(14)60584-5 PMC4441266

[CIT0019] Enomoto M, Endo A, Yatsushige H, Fushimi K, Otomo Y. 2019. Clinical effects of early edaravone use in acute ischemic stroke patients treated by endovascular reperfusion therapy. Stroke. 50(3):652–658. 30741623 10.1161/STROKEAHA.118.023815

[CIT0020] Fisher M, Feuerstein G, Howells DW, Hurn PD, Kent TA, Savitz SI, Lo EH, Group S, STAIR Group 2009. Update of the stroke therapy academic industry roundtable preclinical recommendations. Stroke. 40(6):2244–2250. 19246690 10.1161/STROKEAHA.108.541128 PMC2888275

[CIT0021] Frank-Cannon TC, Alto LT, McAlpine FE, Tansey MG. 2009. Does neuroinflammation fan the flame in neurodegenerative diseases? Mol Neurodegener. 4:47. 19917131 10.1186/1750-1326-4-47 PMC2784760

[CIT0022] Gao Y, Yu WX, Duan XM, Ni LL, Liu H, Zhao HR, Xiao H, Zhang CG, Yang ZB. 2020. Wasp venom possesses potential therapeutic effect in experimental models of rheumatoid arthritis. Evid Based Complement Alternat Med. 2020:6394625. 32328136 10.1155/2020/6394625 PMC7165351

[CIT0023] Giaume C, Koulakoff A, Roux L, Holcman D, Rouach N. 2010. Astroglial networks: a step further in neuroglial and gliovascular interactions. Nat Rev Neurosci. 11(2):87–99. 20087359 10.1038/nrn2757

[CIT0024] Ginsberg MD, Pulsinelli WA. 1994. The ischemic penumbra, injury thresholds, and the therapeutic window for acute stroke. Ann Neurol. 36(4):553–554. 7944286 10.1002/ana.410360402

[CIT0025] Grotta J, Ackerman R, Correia J, Fallick G, Chang J. 1982. Whole blood viscosity parameters and cerebral blood flow. Stroke. 13(3):296–301. 7080121 10.1161/01.str.13.3.296

[CIT0026] Hacke W, Kaste M, Bluhmki E, Brozman M, Davalos A, Guidetti D, Larrue V, Lees KR, Medeghri Z, Machnig T, et al. 2008. Thrombolysis with alteplase 3 to 4.5 hours after acute ischemic stroke. N Engl J Med. 359(13):1317–1329. 18815396 10.1056/NEJMoa0804656

[CIT0027] Hakim AM. 1987. The cerebral ischemic penumbra. Can J Neurol Sci. 14(4):557–559. 2446732

[CIT0028] Herz J, Hagen SI, Bergmuller E, Sabellek P, Gothert JR, Buer J, Hansen W, Hermann DM, Doeppner TR. 2014. Exacerbation of ischemic brain injury in hypercholesterolemic mice is associated with pronounced changes in peripheral and cerebral immune responses. Neurobiol Dis. 62:456–468. 24184800 10.1016/j.nbd.2013.10.022

[CIT0029] Hirt L, Fukuda AM, Ambadipudi K, Rashid F, Binder D, Verkman A, Ashwal S, Obenaus A, Badaut J. 2017. Improved long-term outcome after transient cerebral ischemia in aquaporin-4 knockout mice. J Cereb Blood Flow Metab. 37(1):277–290. 26767580 10.1177/0271678X15623290 PMC5363745

[CIT0030] Hu Y, Zhao H, Lu J, Xie D, Wang Q, Huang T, Xin H, Hisatome I, Yamamoto T, Wang W, et al. 2021. High uric acid promotes dysfunction in pancreatic β cells by blocking IRS2/AKT signalling. Mol Cell Endocrinol. 520:111070. 33127482 10.1016/j.mce.2020.111070

[CIT0031] Jauch EC, Saver JL, Adams HP, Bruno A, Connors JJ, Demaerschalk BM, Khatri P, McMullan PW, Qureshi AI, Rosenfield K, et al. 2013. Guidelines for the early management of patients with acute ischemic stroke: a guideline for healthcare professionals from the American Heart Association/American Stroke Association. Stroke. 44(3):870–947. 23370205 10.1161/STR.0b013e318284056a

[CIT0032] Jin R, Yang G, Li G. 2010. Inflammatory mechanisms in ischemic stroke: role of inflammatory cells. J Leukoc Biol. 87(5):779–789. 20130219 10.1189/jlb.1109766 PMC2858674

[CIT0033] Jones S, Howl J. 2012. Enantiomer-specific bioactivities of peptidomimetic analogues of mastoparan and mitoparan: characterization of inverso mastoparan as a highly efficient cell penetrating peptide. Bioconjug Chem. 23(1):47–56. 22148546 10.1021/bc2002924

[CIT0034] Khalil WK, Assaf N, ElShebiney SA, Salem NA. 2015. Neuroprotective effects of bee venom acupuncture therapy against rotenone-induced oxidative stress and apoptosis. Neurochem Int. 80:79–86. 25481089 10.1016/j.neuint.2014.11.008

[CIT0035] Kim ME, Lee JY, Lee KM, Park HR, Lee E, Lee Y, Lee JS, Lee J. 2016. Neuroprotective effect of bee venom is mediated by reduced astrocyte activation in a subchronic MPTP-induced model of Parkinson's disease. Arch Pharm Res. 39(8):1160–1170. 27469335 10.1007/s12272-016-0802-0

[CIT0036] Kleinschnitz C, Pozgajova M, Pham M, Bendszus M, Nieswandt B, Stoll G. 2007. Targeting platelets in acute experimental stroke. Circulation. 115(17):2323–2330. 17438148 10.1161/CIRCULATIONAHA.107.691279

[CIT0037] Kobayashi S, Fukuma S, Ikenoue T, Fukuhara S, Kobayashi S. 2019. Effect of edaravone on neurological symptoms in real-world patients with acute ischemic stroke. Stroke. 50(7):1805–1811. 31164072 10.1161/STROKEAHA.118.024351

[CIT0038] Krupinski J, Kaluza J, Kumar P, Kumar S, Wang JM. 1994. Role of angiogenesis in patients with cerebral ischemic stroke. Stroke. 25(9):1794–1798. 7521076 10.1161/01.str.25.9.1794

[CIT0039] Kuge Y, Minematsu K, Yamaguchi T, Miyake Y. 1995. Nylon monofilament for intraluminal middle cerebral-artery occlusion in rats. Stroke. 26(9):1655–1657. 7660413 10.1161/01.str.26.9.1655

[CIT0040] Levy EI, Siddiqui AH, Crumlish A, Snyder KV, Hauck EF, Fiorella DJ, Hopkins LN, Mocco J. 2009. First food and drug administration-approved prospective trial of primary intracranial stenting for acute stroke: SARIS (stent-assisted recanalization in acute ischemic stroke). Stroke. 40(11):3552–3556. 19696415 10.1161/STROKEAHA.109.561274

[CIT0041] Li RY, Cao ZG, Li Y, Wang RT. 2015. Increased whole blood viscosity is associated with silent cerebral infarction. Clin Hemorheol Microcirc. 59(4):301–307. 23988733 10.3233/CH-131760

[CIT0042] Liesz A, Hagmann S, Zschoche C, Adamek J, Zhou W, Sun L, Hug A, Zorn M, Dalpke A, Nawroth P, et al. 2009. The spectrum of systemic immune alterations after murine focal ischemia: immunodepression versus immunomodulation. Stroke. 40(8):2849–2858. 19443795 10.1161/STROKEAHA.109.549618

[CIT0043] Lipton P. 1999. Ischemic cell death in brain neurons. Physiol Rev. 79(4):1431–1568. 10508238 10.1152/physrev.1999.79.4.1431

[CIT0044] Liu F, McCullough LD. 2011. Middle cerebral artery occlusion model in rodents: methods and potential pitfalls. J Biomed Biotechnol. 2011:464701. 21331357 10.1155/2011/464701 PMC3035178

[CIT0045] Lo EH, Dalkara T, Moskowitz MA. 2003. Mechanisms, challenges and opportunities in stroke. Nat Rev Neurosci. 4(5):399–414. 12728267 10.1038/nrn1106

[CIT0046] Longa EZ, Weinstein PR, Carlson S, Cummins R. 1989. Reversible middle cerebral artery occlusion without craniectomy in rats. Stroke. 20(1):84–91. 2643202 10.1161/01.str.20.1.84

[CIT0047] Low PC, Manzanero S, Mohannak N, Narayana VK, Nguyen TH, Kvaskoff D, Brennan FH, Ruitenberg MJ, Gelderblom M, Magnus T, et al. 2014. PI3Kδ inhibition reduces TNF secretion and neuroinflammation in a mouse cerebral stroke model. Nat Commun. 5(1):12. 10.1038/ncomms4450 24625684

[CIT0048] Moon DO, Park SY, Lee KJ, Heo MS, Kim KC, Kim MO, Lee JD, Choi YH, Kim GY. 2007. Bee venom and melittin reduce proinflammatory mediators in lipopolysaccharide-stimulated BV2 microglia. Int Immunopharmacol. 7(8):1092–1101. 17570326 10.1016/j.intimp.2007.04.005

[CIT0049] Moreau ME, Garbacki N, Molinaro G, Brown NJ, Marceau F, Adam A. 2005. The kallikrein-kinin system: current and future pharmacological targets. J Pharmacol Sci. 99(1):6–38. 16177542 10.1254/jphs.srj05001x

[CIT0050] Mortari MR, Cunha AO, de Oliveira L, Vieira EB, Gelfuso EA, Coutinho-Netto J, Ferreira dos Santos W. 2005. Anticonvulsant and behavioural effects of the denatured venom of the social wasp *Polybia occidentalis* (Polistinae, Vespidae). Basic Clin Pharmacol Toxicol. 97(5):289–295. 16236140 10.1111/j.1742-7843.2005.pto_137.x

[CIT0051] Moskowitz MA, Lo EH, Iadecola C. 2010. The science of stroke: mechanisms in search of treatments. Neuron. 67(2):181–198. 20670828 10.1016/j.neuron.2010.07.002 PMC2957363

[CIT0052] Nayak D, Roth TL, McGavern DB. 2014. Microglia development and function. Annu Rev Immunol. 32(1):367–402. 24471431 10.1146/annurev-immunol-032713-120240 PMC5001846

[CIT0053] Nitta T, Hata M, Gotoh S, Seo Y, Sasaki H, Hashimoto N, Furuse M, Tsukita S. 2003. Size-selective loosening of the blood-brain barrier in claudin-5–deficient mice. J Cell Biol. 161(3):653–660. 12743111 10.1083/jcb.200302070 PMC2172943

[CIT0054] Noda M, Kariura Y, Pannasch U, Nishikawa K, Wang L, Seike T, Ifuku M, Kosai Y, Wang B, Nolte C, et al. 2007. Neuroprotective role of bradykinin because of the attenuation of pro-inflammatory cytokine release from activated microglia. J Neurochem. 101(2):397–410. 17402969 10.1111/j.1471-4159.2006.04339.x

[CIT0055] O-Uchi T, Tanaka Y. 1988. Study of the so-called cochlear mechanical tinnitus. Acta Oto-Laryngol. 105(447):94–99. 10.3109/00016488809102863 3188900

[CIT0056] Pan J, Konstas AA, Bateman B, Ortolano GA, Pile-Spellman J. 2007. Reperfusion injury following cerebral ischemia: pathophysiology, MR imaging, and potential therapies. Neuroradiology. 49(2):93–102. 17177065 10.1007/s00234-006-0183-z PMC1786189

[CIT0057] Pereda AE. 2014. Electrical synapses and their functional interactions with chemical synapses. Nat Rev Neurosci. 15(4):250–263. 24619342 10.1038/nrn3708 PMC4091911

[CIT0058] Perry VH, Nicoll JAR, Holmes C. 2010. Microglia in neurodegenerative disease. Nat Rev Neurol. 6(4):193–201. 20234358 10.1038/nrneurol.2010.17

[CIT0059] Rádis-Baptista G. 2021. Cell-penetrating peptides derived from animal venoms and toxins. Toxins. 13(2):147. 33671927 10.3390/toxins13020147 PMC7919042

[CIT0060] Ren X, Hu H, Farooqi I, Simpkins JW. 2020. Blood substitution therapy rescues the brain of mice from ischemic damage. Nat Commun. 11(1):4078. 32843630 10.1038/s41467-020-17930-x PMC7447645

[CIT0061] Saba E, Shafeeq T, Irfan M, Lee YY, Kwon HW, Seo MG, Park SJ, Lee KY, Rhee MH. 2017. Anti-inflammatory activity of crude venom isolated from parasitoid wasp, bracon hebetor say. Mediat Inflamm. 2017:6978194. 10.1155/2017/6978194 PMC5682083 29213193

[CIT0062] Shichita T, Hasegawa E, Kimura A, Morita R, Sakaguchi R, Takada I, Sekiya T, Ooboshi H, Kitazono T, Yanagawa T, et al. 2012. Peroxiredoxin family proteins are key initiators of post-ischemic inflammation in the brain. Nat Med. 18(6):911–917. 22610280 10.1038/nm.2749

[CIT0063] Shichita T, Sugiyama Y, Ooboshi H, Sugimori H, Nakagawa R, Takada I, Iwaki T, Okada Y, Iida M, Cua DJ, et al. 2009. Pivotal role of cerebral interleukin-17-producing gammadelta T cells in the delayed phase of ischemic brain injury. Nat Med. 15(8):946–950. 19648929 10.1038/nm.1999

[CIT0064] Simard JM, Kent TA, Chen M, Tarasov KV, Gerzanich V. 2007. Brain oedema in focal ischaemia: molecular pathophysiology and theoretical implications. Lancet Neurol. 6(3):258–268. 17303532 10.1016/S1474-4422(07)70055-8 PMC2725365

[CIT0065] Song YM. 2011. Topographic patterns of thalamic infarcts in association with stroke syndromes and aetiologies. J Neurol Neurosurg Psychiatry. 82(10):1083–1086. 21406535 10.1136/jnnp.2010.239624

[CIT0066] Sternberg EM. 2006. Neural regulation of innate immunity: a coordinated nonspecific host response to pathogens. Nat Rev Immunol. 6(4):318–328. 16557263 10.1038/nri1810 PMC1783839

[CIT0067] Su Y, Zhang L, Zhou Y, Ding L, Li L, Wang Z. 2021. The progress of research on histone methylation in ischemic stroke pathogenesis. J Physiol Biochem. DOI: 10.1007/s13105-021-00841-w 34472033

[CIT0068] Thathiah A, De Strooper B. 2011. The role of G protein-coupled receptors in the pathology of Alzheimer’s disease. Nat Rev Neurosci. 12(2):73–87. 21248787 10.1038/nrn2977

[CIT0069] Tobin MK, Bonds JA, Minshall RD, Pelligrino DA, Testai FD, Lazarov O. 2014. Neurogenesis and inflammation after ischemic stroke: what is known and where we go from here. J Cereb Blood Flow Metab. 34(10):1573–1584. 25074747 10.1038/jcbfm.2014.130 PMC4269726

[CIT0070] Turner RC, Dodson SC, Rosen CL, Huber JD. 2013. The science of cerebral ischemia and the quest for neuroprotection: navigating past failure to future success. J Neurosurg. 118(5):1072–1085. 23331000 10.3171/2012.11.JNS12408 PMC4652647

[CIT0071] Wang Y, Hayashi T, Chang CF, Chiang YH, Tsao LI, Su TP, Borlongan C, Lin SZ. 2001. Methamphetamine potentiates ischemia/reperfusion insults after transient middle cerebral artery ligation. Stroke. 32(3):775–782. 11239201 10.1161/01.str.32.3.775

[CIT0072] Warach S, Latour LL. 2004. Evidence of reperfusion injury, exacerbated by thrombolytic therapy, in human focal brain ischemia using a novel imaging marker of early blood-brain barrier disruption. Stroke. 35(1):2659–2661. 15472105 10.1161/01.STR.0000144051.32131.09

[CIT0073] Wardlaw JM, Murray V, Berge E, del Zoppo G, Sandercock P, Lindley RL, Cohen G. 2012. Recombinant tissue plasminogen activator for acute ischaemic stroke: an updated systematic review and meta-analysis. Lancet. 379(9834):2364–2372. 22632907 10.1016/S0140-6736(12)60738-7 PMC3386494

[CIT0074] Wu S, Wu B, Liu M, Chen Z, Wang W, Anderson CS, Sandercock P, Wang Y, Huang Y, Cui L, et al. 2019. Stroke in China: advances and challenges in epidemiology, prevention, and management. Lancet Neurol. 18(4):394–405. 30878104 10.1016/S1474-4422(18)30500-3

[CIT0075] Xu AD, Wang YJ, Wang DZ, Expert CST. 2013. Consensus statement on the use of intravenous recombinant tissue plasminogen activator to treat acute ischemic stroke by the Chinese stroke therapy expert panel. CNS Neurosci Ther. 19(8):543–548. 23710819 10.1111/cns.12126 PMC6493470

[CIT0076] Yang EJ, Jiang JH, Lee SM, Yang SC, Hwang HS, Lee MS, Choi SM. 2010. Bee venom attenuates neuroinflammatory events and extends survival in amyotrophic lateral sclerosis models. J Neuroinflammation. 7:69. 20950451 10.1186/1742-2094-7-69 PMC2974667

[CIT0077] Zhang XM, Du F, Yang D, Yu CJ, Huang XN, Liu W, Fu J. 2010. Transplanted bone marrow stem cells relocate to infarct penumbra and co-express endogenous proliferative and immature neuronal markers in a mouse model of ischemic cerebral stroke. BMC Neurosci. 11:138. 20973978 10.1186/1471-2202-11-138 PMC2974740

[CIT0078] Zhang YM, Qu XY, Tao LN, Zhai JH, Gao H, Song YQ, Zhang SX. 2020. XingNaoJing injection ameliorates cerebral ischaemia/reperfusion injury via SIRT1-mediated inflammatory response inhibition. Pharm Biol. 58(1):16–24. 31854225 10.1080/13880209.2019.1698619 PMC6968491

[CIT0079] Zhang ZG, Chopp M. 2015. Promoting brain remodeling to aid in stroke recovery. Trends Mol Med. 21(9):543–548. 26278490 10.1016/j.molmed.2015.07.005 PMC4567429

[CIT0080] Zhang ZG, Zhang L, Tsang W, Soltanian-Zadeh H, Morris D, Zhang R, Goussev A, Powers C, Yeich T, Chopp M. 2002. Correlation of VEGF and angiopoietin expression with disruption of blood-brain barrier and angiogenesis after focal cerebral ischemia. J Cereb Blood Flow Metab. 22(4):379–392. 11919509 10.1097/00004647-200204000-00002

[CIT0081] Zhou ST, Luan K, Ni LL, Wang Y, Yuan SM, Che YH, Yang ZZ, Zhang CG, Yang ZB. 2019. A strategy for quality control of *vespa magnifica* (Smith) venom based on HPLC fingerprint analysis and multi-component separation combined with quantitative analysis. Molecules. 24(16):2920. 10.3390/molecules24162920 PMC6719070 31408988

