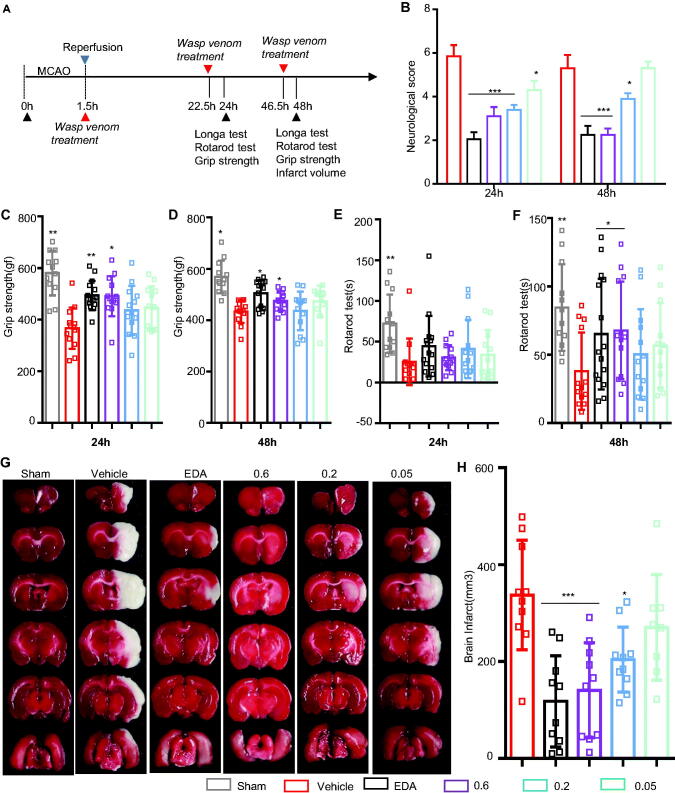# Correction

**DOI:** 10.1080/13880209.2022.2130609

**Published:** 2022-10-06

**Article title:** Wasp venom from *Vespa magnifica* acts as a neuroprotective agent to alleviate neuronal damage after stroke in rats

**Authors:** Hairong Zhao, Mei Wang, Xi Huang, Xiumei Wu, Huai Xiao, Fanmao Jin, Jiaming Lv, Jidong Cheng, Yu Zhao and Chenggui Zhang

**Journal:** *Pharmaceutical Biology*

**Bibliometrics:** Volume 60, Number 01, pages 334–346

**DOI:** http://dx.doi.org/10.1080/13880209.2022.2032207

When originally published, this paper contained an incorrect figure. Figure 2 has now been updated with the correct figure.